# Repair of the complete atrioventricular septal defect—impact of postoperative moderate or more regurgitation

**DOI:** 10.1093/icvts/ivae053

**Published:** 2024-04-03

**Authors:** Mahmut Ozturk, Aybala Tongut, Vanessa Sterzbecher, Manan Desai, Gabriel Esmailian, Soichiro Henmi, Christopher Spurney, Steven J Staffa, Yves d’Udekem, Can Yerebakan

**Affiliations:** Division of Cardiac Surgery, Children’s National Hospital, The George Washington University School of Medicine and Health Sciences, Washington, DC, USA; Division of Cardiac Surgery, Children’s National Hospital, The George Washington University School of Medicine and Health Sciences, Washington, DC, USA; Division of Cardiac Surgery, Children’s National Hospital, The George Washington University School of Medicine and Health Sciences, Washington, DC, USA; Division of Cardiac Surgery, Children’s National Hospital, The George Washington University School of Medicine and Health Sciences, Washington, DC, USA; Division of Cardiac Surgery, Children’s National Hospital, The George Washington University School of Medicine and Health Sciences, Washington, DC, USA; Division of Cardiac Surgery, Children’s National Hospital, The George Washington University School of Medicine and Health Sciences, Washington, DC, USA; Division of Cardiology, Children’s National Hospital, The George Washington University School of Medicine and Health Sciences, Washington, DC, USA; Department of Anesthesiology, Critical Care and Pain Medicine, Boston Children's Hospital, Harvard Medical School, Boston, MA, USA; Division of Cardiac Surgery, Children’s National Hospital, The George Washington University School of Medicine and Health Sciences, Washington, DC, USA; Division of Cardiac Surgery, Children’s National Hospital, The George Washington University School of Medicine and Health Sciences, Washington, DC, USA

**Keywords:** Atrioventricular septal defect, Double patch technique, Australian single patch technique, Left atrioventricular valve regurgitation

## Abstract

**OBJECTIVES:**

To study the risk factors for mortality, moderate or more left atrioventricular valve regurgitation (LAVVR) and reoperation after the surgical repair of complete atrioventricular septal defect (cAVSD) in a single centre.

**METHODS:**

The current study is a retrospective review of patients who underwent surgical repair of cAVSD between 2000 and 2021. Patients with unbalanced ventricles not amenable to biventricular repair, double outlet right ventricle and malpositioned great arteries were excluded. The clinical predictors of outcome for end points were analysed with univariate and multivariable Cox regression analysis or Fine–Gray modelling for competing risks. Time-dependent end points were estimated using the Kaplan–Meier curve analysis and cumulative incidence curves.

**RESULTS:**

The median follow-up time was 2.3 years. Among 220 consecutive patients were 10 (4.6%) operative and 21 late mortalities (9.6%). A total of 26 patients were identified to have immediate postoperative moderate or more regurgitation and 10 of them ultimately died. By multivariable analysis prematurity and having more than moderate regurgitation immediately after the operation were identified as predictors of overall mortality (*P* = 0.003, *P* = 0.012). Five- and ten-year survival rates were lower for patients with immediate postoperative moderate or more LAVVR {51.9% [confidence interval (CI): 27.5–71.7%]} when compared to patients without moderate or more regurgitation [93.2% (CI: 87.1–96.4%) and 91.3% (CI: 83.6–95.5%)].

**CONCLUSIONS:**

The patients who undergo cAVSD repair remain subjected to a heavy burden of disease related to postoperative residual LAVVR. Immediate postoperative moderate or more LAVVR contributes significantly to overall mortality. Whether a second run of bypass can decrease this observed mortality should be investigated.

**Figure:**
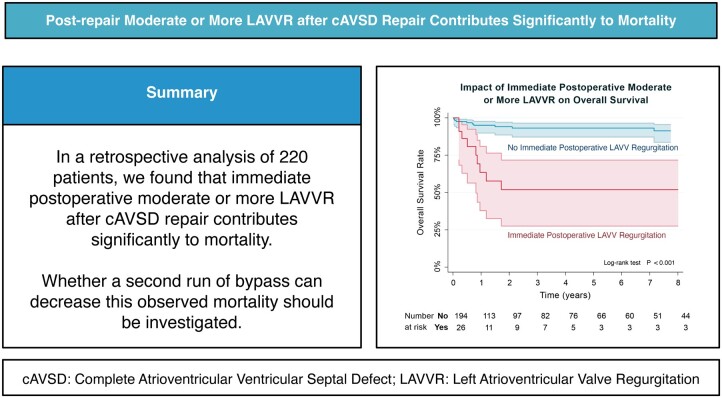

## INTRODUCTION

Patients undergoing repair of complete atrioventricular septal defect (cAVSD) still face a significant risk of reoperation up to 12% within 10 years of the repair most frequently due to moderate or more left atrioventricular valve regurgitation (LAVVR) [1–3]. The debate on the best cAVSD repair remains mostly on the superiority of the 2 dominant surgical techniques -double patch and the Australian single patch- over each other rather than delineating the risk factors for undesirable outcome. Recent studies suggest the equivalence of these techniques in terms of reoperation [4, 5]. However, predictors of adverse events following surgical cAVSD repair remain obscure.

Having a substantial number of cAVSD patients, we conducted a retrospective single-centre study, where 3 different surgical patch techniques for cAVSD repair were utilized by 12 different surgeons over a 21-year period to identify risk factors for operative and overall mortality as well as reoperations due to residual LAVVR.

## PATIENTS AND METHODS

### Ethical statement

The study was approved by the Institutional Review Board at Children's National Hospital (IRB # PRO00015566) and the requirement for individual patient consent was waived.

The patients who underwent cAVSD repair between the years of 2000–2021 were included in the study. Those with unbalanced atrioventricular septal defect (AVSD) not amenable to biventricular repair, double outlet right ventricle, malpositioned great arteries were excluded. During the study period, 12 surgeons from our department performed all cAVSD repairs. The patch technique, cleft closure and fenestration of the interatrial septum were based on surgeon’s preference. (See Supplementary Material for a brief explanation of surgical techniques.)

Operative mortality was defined according to the definition by the Society of Thoracic Surgeons (STS) Congenital Heart Surgery Database [6] as mortality during the same hospitalization even if after 30 days or within the 30 days after the surgery.

Follow-up data were collected from medical records and institutional databases. Echocardiographic data were acquired from individual echocardiographic assessments, in collaboration with the cardiologists.

The evaluation of mitral valve regurgitation was performed retrospectively on clinical imaging and necessitated qualitative assessment. Mild regurgitation was assessed by colour Doppler as a small, central and often brief signal while severe regurgitation included a large central jet, often >50% of left atrium, or an eccentric wall-impinging jet of variable size. Using continuous wave spectral Doppler, a mild signal was faint or partial and parabolic while a severe regurgitation signal was dense, holosystolic, truncated and triangular. Moderate mitral regurgitation was determined as greater than mild but did not exhibit the described characteristics of severe regurgitation [7].

The immediate postoperative grading of the LAVVR quoted to be mild-to-moderate were revisited by an independent cardiologist. Left ventricular outflow tract obstruction (LVOTO) was defined as a gradient of >20 mmHg across the left ventricular outflow tract or any LVOTO requiring surgical relief. The patients born before 37th gestational week were classified as premature.

### Study end points

The primary outcomes of the study were operative mortality after the index operation and the long-term survival as well as freedom from moderate or more LAVVR and/or freedom from reoperation due to moderate or more LAVVR. Length of hospital stay was analysed as a secondary outcome.

### Statistical analysis

Statistical analysis was performed using STATA software version 16.1 (StataCorp LLC, College Station, TX, USA). Categorical variables are expressed using frequencies and percentages. Continuous data were expressed as medians and interquartile range. The Shapiro–Wilk test was used to indicate significant deviation from the normal distribution. With the exception of placement of coronary sinus, there was no missing data. All available data were analysed, and a complete case analysis was implemented for multivariable analysis. For each patient, the follow-up index was calculated as the proportion of actual follow-up period for each patient versus the maximum possible follow-up period. Freedom from mortality was estimated using the Kaplan–Meier curve analysis, with numbers at risk displayed and 95% confidence intervals obtained using Greenwood’s formula. The log-rank test was implemented to compare survival functions throughout follow-up. Kaplan–Meier curves were estimated for all time-to-event end points as a cause-specific analysis for the event of interest. Categorical variables were tested with Pearson's Chi-squared test and Fisher's exact test. The clinical predictors of mortality were analysed with univariate and multivariable Cox regression analysis, where the risk factors with *P* < 0.1 upon univariate analyses were included in the multivariable model. A statistical interaction term for left atrioventricular valve (LAVV) reoperation and time to LAVV reoperation was evaluated. The results of the Cox regression models are reported as adjusted hazard ratios with a 95% confidence interval (CI) and *P*-values. Competing risks analysis using the Fine–Gray model was implemented for univariate and multivariable analysis of LAVV reoperation and postoperative moderate LAVVR, with mortality included as a competing event since mortality may preclude these events. Results from Fine–Gray competing risk modelling are presented as adjusted subdistribution hazard ratios with 95% confidence intervals and *P*-values. Cumulative incidence curves were created for LAVV reoperation and postoperative moderate LAVVR. Continuous outcomes including the length of hospital stay were assessed with univariate and multivariable median regression analysis. Cross-clamp time was categorized based on quartiles of its distribution for regression modelling. Again, the risk factors with *P* < 0.1 upon univariate analyses were included in the multivariable model. Unless otherwise stated, univariate regression *P*-value was reported next to each variable in the results section. A two-tailed *P* < 0.05 was implemented to determine statistical significance.

## RESULTS

### Characteristics

Between 2000 and 2021, a total of 390 patients underwent biventricular surgical repair of AVSD at the Children's National Hospital, Washington, DC. Repair of cAVSD was performed in 220 patients. The baseline characteristics of the cohort are summarized in Table 1. The Median follow-up was 28 months (6–87). The median follow-up index for patients in the cohort was 0.72 (0.13–0.98). The median gestational age was 38 (36–40) weeks. The number of patients born prematurely was 41 (18.6%). The median birth weight (kg) was 2.9 (2.3–3.3). A total of 183 (83.2%) patients were diagnosed with a genetic condition. Trisomy 21 was diagnosed in 166 (75.5%) patients. Twenty-one patients (9.5%) had moderate or more LAVVR in preoperative transoesophageal echocardiography. Moderate or more right atrioventricular valve regurgitation was diagnosed in 22 (10.0%) patients prior to surgery. As per analysis of an independent cardiologist, 13 of the 24 patients with immediate post-operative mild-to-moderate regurgitation were reclassified as having moderate regurgitation and 11 mild. Between the years of 2000–2004, 2005–2009, 2010–2014 and 2015–2021, 4 (1.8%), 68 (30.9%), 57 (25.9%) and 90 (40.9%) patients were operated respectively.

**Table 1:** Patient characteristics.

	Overall, *n* (%) or median (IQR)
Total number of patients	220
Gestational age (weeks)	38 (36–40)
Gender	
Female	130 (59.1%)
Male	90 (40.1%)
Prematurity	41 (18.6%)
Birth weight (kg)	2.9 (2.3–3.3)
Genetic syndrome	183 (83.2%)
Trisomy 21	166 (75.5%)
Heterotaxy	3 (1.4%)
History of coarctation repair	6 (2.7%)
History of pulmonary artery banding	17 (7.7%)

IQR: interquartile range.

### Operative variables

The operative characteristics of the cohort are summarized in Table 2. Median age and weight at surgery were 3.0 (2.6–5.1) months and 4.6 (4.0–5.5) kg. At surgeons' discretion, 166 (75.5%) patients were operated on with the Australian single-patch technique, 48 (21.8%) patients with the double patch, and 6 patients (2.7%) with the traditional single patch technique. The atrial septum was fenestrated in 61 (27.7%) patients and a patent ductus arteriosus was ligated in 46 (20.9%) patients. Total cleft closure was performed in 211 (95.6%) patients. Immediate postoperative transoesophageal echocardiography demonstrated moderate or more LAVVR in 26 (11.8%) patients. Eight (3.6%) patients required extracorporeal membrane oxygenation support following the initial repair. Four of these patients (50%) could not be discharged.

**Table 2:** Operative characteristics

	Overall, *n* (%) or median (IQR)
Patch technique	
Double patch	48 (21.8%)
Australian single patch	166 (75.5%)
Traditional single patch	6 (2.7%)
Rastelli classification	
Rastelli A	137 (62.2%)
Rastelli B	1 (0.5%)
Rastelli C	82 (37.3%)
Weight at surgery (kg)	4.6 (4.0–5.5)
Age at surgery (months)	3.0 (2.6–5.1)
Cleft closure	211 (95.6%)
Hospital stay (days)	10 (6.0–21.3)
Cross-clamp time (min)	62.5 (47–87)

IQR: interquartile range.

### Mortality

#### Operative mortality

There were 10 (4.6%) operative mortalities after cAVSD repair, of whom 2 of 48 (4.2%) had received a double patch technique and 8 of 166 (4.8%) Australian single patch technique (*P* = 0.85). All the patients operated with a traditional single patch (*n* = 6) survived. Three out of the 10 patients who died (30%) had immediate postoperative moderate or more LAVV regurgitation (*P* = 0.09). The median time to operative mortality was 77 (24–179) days. The median time to operative mortality for the double patch and the Australian single patch patients was 18.8 (16–21) and 96.5 (68–179) days, respectively. Four of the 10 mortalities (40%) were born premature (*P* = 0.09). One of the 5 patients who were operated on below 2.5 kg died (*P* = 0.14). Two of 21 patients (9.5%) who had moderate or more LAVV regurgitation prior to surgery died (*P* = 0.27). There were no difference noted in the mortality faced by individual surgeons (*P* = 0.78). Multivariable logistics regression analysis did not identify any significant preoperative factor for operative mortality. (See Supplementary Material, Table S1 which depicts the characteristics of the patients who died within 30 days or before discharge after cAVSD repair.)

#### Overall mortality

Overall, there were 21 (9.6%) mortalities after cAVSD repair. Ten of these patients (47.6%) had immediate postoperative moderate or more regurgitation of the LAVV (Cox regression *P* < 0.001). Survival was significantly lower for patients with immediate postoperative moderate or more LAVVR (Log-rank test *P* < 0.001). Five- and ten-year survival rates for patients with immediate postoperative moderate or more LAVVR were both 51.9% (CI: 27.5–71.7%) as compared to 93.2% (CI: 87.1–96.4%) and 88% (CI: 77.8–94.2%) in patients without moderate or more regurgitation.

Ten of the 26 (38.5%) patients who were identified to have moderate or more regurgitation on the immediate post-operative echocardiography ultimately died (Cox regression *P* < 0.001).

**Figure 1:**
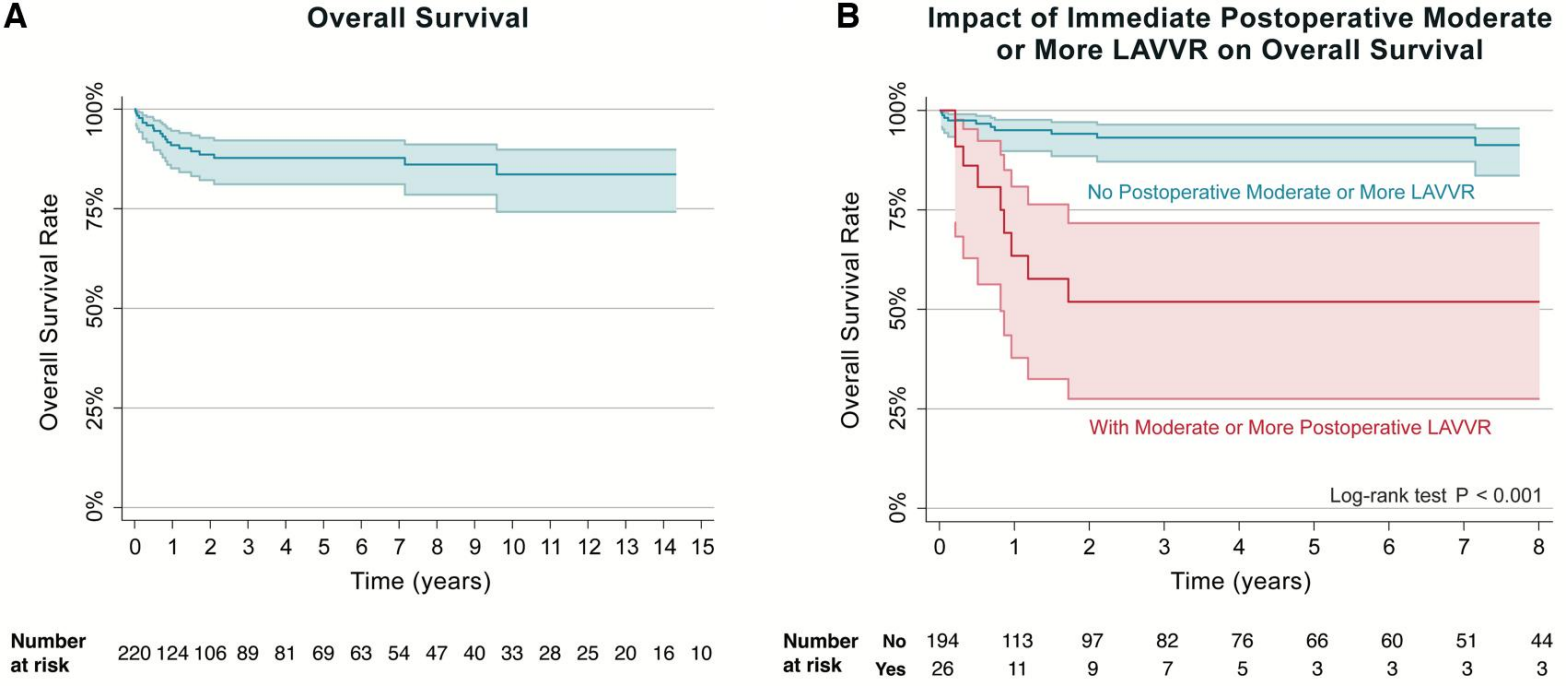
Kaplan–Meier curves depicting long-term survival for all patients (left) and the comparison of survival rates by immediate postoperative moderate or greater LAVV regurgitation (right). LAVV: left atrioventricular valve.

Multivariable Cox regression analysis identified prematurity (*P* = 0.003) and immediate postoperative moderate or more LAVV regurgitation (*P* = 0.012) as significant risk factors for mortality after cAVSD repair (Fig. 1 and Tables 3 and 4).

**Table 3:** Univariate analysis of predictors of overall mortality

Variable	HR	95% CI	*P-*value
Surgical age (days)	1.0 per month	(0.99,1.01)	0.802
Surgical age <6 months	1.12	(0.33,3.84)	0.849
Prematurity	5.51	(2.26,13.4)	<0.001^*^
Gender			
Female	1.43	(0.57,3.54)	0.444
Male	Reference	.	.
Weight (kg)	1.01 per kg	(0.94,1.08)	0.804
Weight <2.5 kg	2.84	(0.38,21.4)	0.31
Cleft closure	Cannot calculate	.	0.392
Patch technique			
Double patch	Reference	.	.
Australian single patch	1.92	(0.44,8.35)	0.387
Traditional single patch	4.74	(0.66,33.9)	0.121
Down syndrome	1.11	(0.37,3.31)	0.85
Any syndrome	1.42	(0.42,4.84)	0.573
Moderate or more LAVVR prior to surgery	1.47	(0.34,6.34)	0.608
Moderate or more RAVVR prior to surgery	1.66	(0.49,5.66)	0.418
Immediate postoperative moderate LAVVR	7.4	(3.11,17.5)	<0.001^*^
Moderate or more LAVVR at discharge	1.86	(0.72,4.8)	0.2
Moderate or more LAVVR at any time on follow-up	2.95	(1.08,8.07)	0.035^*^
Any cardiac reoperation	2.97	(1.25,7.02)	0.013^*^
LAVV reoperation	3.12	(1.31,7.43)	0.01^*^
LAVV reoperation × time to LAVV reoperation	0.29	(0.05,1.76)	0.18
Placement of coronary sinus			
Left	Reference	.	.
Right	1.42	(0.32,6.36)	0.645
Interatrial septum fenestration	0.73	(0.27,2)	0.542
Cross-clamp time quartile			
Quartile 1 (<47 min)	Reference		
Quartile 2 (47 to <62 min)	3	(0.34,26.9)	0.326
Quartile 3 (62 to <87 min)	6.56	(0.83,51.9)	0.075
Quartile 4 (≥87 min)	6.93	(0.85, 56.8)	0.071
Surgical era			
2000–2004	0.45	(0.04,4.82)	0.513
2005–2009	0.35	(0.09,1.31)	0.119
2010–2014	1.17	(0.43,3.19)	0.752
2015–2021	Reference	.	.

Cox regression analysis was implemented to obtain hazard ratios, 95% CIs and *P*-values. For variables where the hazard ratio cannot be calculated, the log-rank test was implemented.

* Statistically significant.

CI: confidence interval; HR: hazard ratio; LAVV: left atrioventricular valve; LAVVR: Left atrioventricular valve regurgitation; RAVVR: right atrioventricular valve regurgitation.

**Table 4:** Multivariable Cox regression analysis of predictors of overall mortality

Covariate	Adjusted HR	95% CI	*P-*value
Prematurity	4.45	(1.65, 12)	0.003^*^
Immediate postoperative moderate LAVVR	4.02	(1.37, 11.9)	0.012^*^
Moderate or more LAVVR at any time on follow-up	1.49	(0.38, 5.87)	0.568
Any cardiac reoperation	1.77	(0.66, 4.78)	0.257
LAVV reoperation	Omitted due to collinearity with reoperation		
Cross-clamp time quartile			
Quartile 1 (<47 min)	Reference		
Quartile 2 (47 to <62 min)	3.63	(0.37, 35.2)	0.266
Quartile 3 (62 to <87 min)	5.01	(0.6, 41.7)	0.136
Quartile 4 (≥87 min)	7.21	(0.81, 63.8)	0.076

Cox regression analysis was implemented to obtain hazard ratios, 95% CIs and *P*-values. For variables where the hazard ratio cannot be calculated, the log-rank test was implemented.

* Statistically significant.

CI: confidence interval; HR: hazard ratio; LAVV: left atrioventricular valve; LAVVR: Left atrioventricular valve regurgitation.

Two of the 48 (4.2%) patients who underwent repair with the double patch technique, 17 of 166 (10.2%) patients after the repair with the Australian single patch technique and 2 of the 6 (33%) patients after the repair with the traditional single patch technique died.

Overall survival for all patients who underwent cAVSD repair was 87.7% (95% CI: 81.1–92.1%) and 83.6% (95% CI: 74.2–89.8%) at 5 and 10 years. The median time to mortality was 270 (8–5363) days. Overall survival after cAVSD repair with the double patch technique was 95.1% (95% CI: 81.8–98.8%) at 5 and 10 years. For the patients who underwent repair with the Australian single patch survival was lower at 87.5% and 83.0% (95% CI: 79.7–92.4%) at 5 and 10 years (*P* = 0.38). Those underwent repair with the traditional single patch technique had a 62.5% (95% CI: 14.2–89.3%) survival rate during the same period (*P* = 0.121). The median time to mortality after double patch, Australian single patch and the traditional single patch repairs were 18.8 (16–21), 270 (116–546) and 489 (420–558) days, respectively.

### Left atrioventricular valve reoperation

Thirty-two (14.6%) patients underwent LAVV reoperations after cAVSD repair. Immediate postoperative moderate or more LAVVR was significantly associated with LAVV reoperation (*P* = 0.009). Of the 32 patients, 3 (9.4%) had initially a repair with the double patch technique, 28 (87.5%) with the Australian single patch technique (*P* = 0.320) and 1 (3.1%) with the traditional single patch technique (*P* = 0.86). LAVV valvuloplasty was performed in 27 (84.4%) patients, 5 (15.6%) patients underwent mechanical valve replacement and 4 of these 5 patients died eventually. Of the 26 patients with immediate postoperative moderate or more LAVVR, 4 (15.4%) underwent an LAVVR reoperation prior to discharge and all 4 died eventually.

The median time to LAVV reoperation was 276.5 (74–1582) days. The median time to LAVV reoperation after the repair with double patch, Australian single patch and traditional single patch techniques were 922 (593–1695), 265 (68–1582) and 186.7 days, respectively. There was no difference in median times to LAVV reoperation between techniques (*P* = 0.39).

The cumulative incidence estimates for LAVV reoperation for patients who underwent cAVSD repair were 0.2 (95% CI: 0.13, 0.30) and 0.34 (95% CI: 0.23–0.53) at 5 and 10 years.

The cumulative incidence for LAVV reoperation at 5 and 10 years after double patch repair was 0.12 (95% CI: 0.03–0.50) and 0.45 (95% CI: 0.10–2.0). After repair with the Australian single patch technique, it was 0.22 (95% CI: 0.14–0.34) and 0.36 (95% CI: 0.23–0.56) (*P* = 0.29).

**Figure 2:**
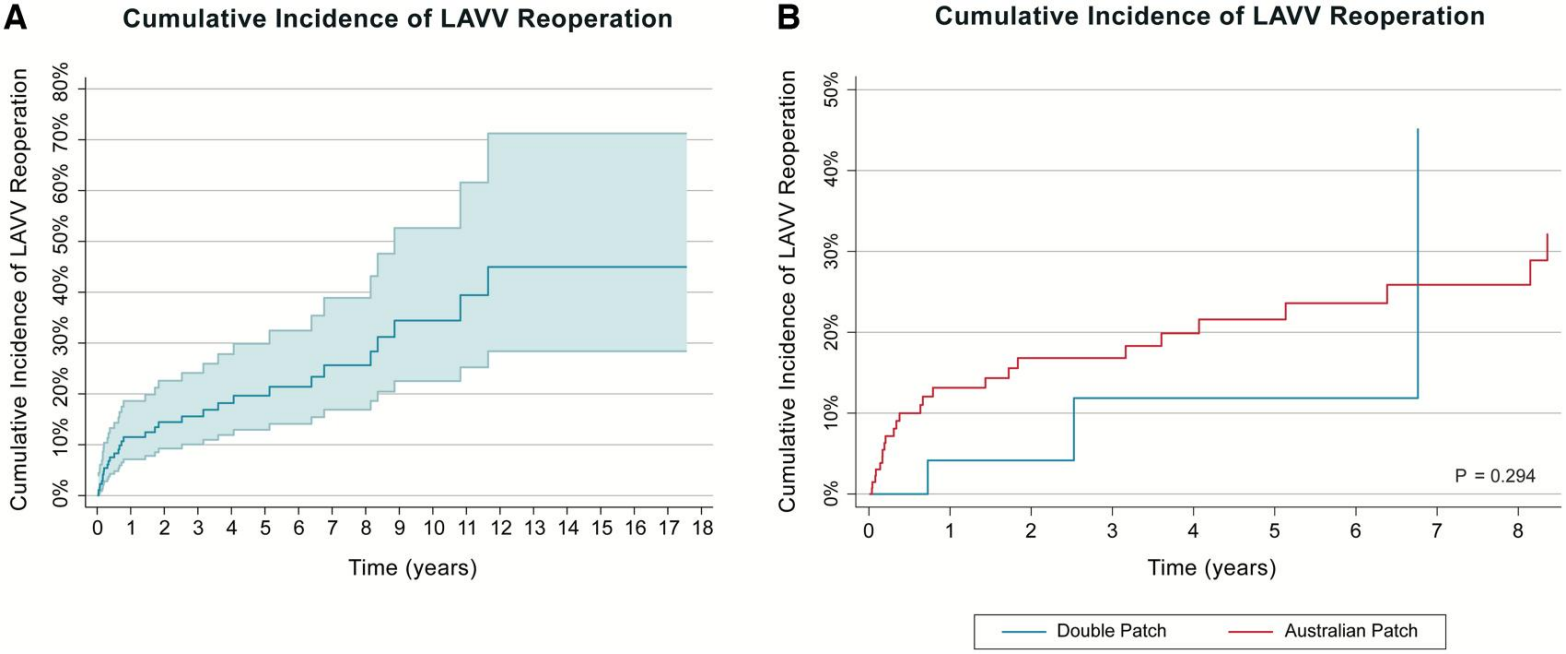
Cumulative incidence curves depicting LAVV reoperation for all patients (left) and the comparison of cumulative incidence of LAVV reoperation rates for the double patch and the Australian single patch techniques (right). LAVV: left atrioventricular valve.

In multivariable competing risk regression analysis using the Fine–Gray model, having immediate postoperative moderate or more postoperative LAVVR was the only predictor of requiring an LAVV reoperation after cAVSD repair (*P* = 0.009) (Fig. 2 and Tables 5 and 6).

**Table 5:** Univariate competing risks analysis of predictors of left atrioventricular valve reoperation using the Fine–Gray model

Variable	sHR	95% CI	*P-*value
Surgical age (days)	0.95 per month	(0.88, 1.04)	0.265
Prematurity	1.84	(0.85, 3.99)	0.121
Gender			
Female	0.8	(0.4, 1.51)	0.534
Male	Reference	–	–
Weight (kg)	0.92 per kg	(0.82, 1.04)	0.18
Weight <2.5 kg	Cannot calculate	–	–
Cleft closure	0.53	(0.14, 2.04)	0.355
Patch technique			
Double patch	Reference	–	–
Australian single patch	1.92	(0.59, 6.28)	0.281
Traditional single patch	1.24	(0.12, 13.1)	0.861
Down syndrome	1.15	(0.47, 2.79)	0.762
Any syndrome	1.01	(0.41, 2.45)	0.991
Moderate or more LAVVR prior to surgery	1.47	(0.46, 4.71)	0.518
Moderate or more RAVVR prior to surgery	1.16	(0.34, 3.88)	0.813
Immediate postoperative moderate LAVVR	3.15	(1.33, 7.5)	0.009^*^
Moderate or more LAVVR at discharge	2.62	(1.23, 5.58)	0.012^*^
Moderate or more LAVVR at any time on follow-up	Cannot calculate		
Placement of coronary sinus			
Left	Reference	–	–
Right	0.32	(0.04, 2.31)	0.258
Interatrial septum fenestration	0.82	(0.39, 1.76)	0.619
Cross-clamp time quartile			
Quartile 1 (<47 min)	Reference		
Quartile 2 (47 to <62 min)	0.73	(0.28, 1.87)	0.512
Quartile 3 (62 to <87 min)	0.88	(0.37, 2.12)	0.777
Quartile 4 (≥87 min)	0.58	(0.2, 1.68)	0.311
Surgical era			
2000–2004	0.8	(0.08, 8.52)	0.853
2005–2009	0.88	(0.34, 2.28)	0.793
2010–2014	1.53	(0.61, 3.8)	0.363
2015–2021	Reference	–	–

Fine–Gray regression analysis was implemented to obtain hazard ratios, 95% CIs and *P*-values.

* Statistically significant.

CI: confidence interval; LAVVR: Left atrioventricular valve regurgitation; RAVVR: right atrioventricular valve regurgitation; sHR: subdistribution hazard ratio.

**Table 6:** Multivariable competing risk regression analysis of predictors of reoperation left atrioventricular valve using the Fine–Gray model

Covariate	Adjusted sHR	95% CI	*P*-value
Immediate postoperative moderate LAVVR	3.15	(1.33, 7.5)	0.009^*^
Moderate or more LAVVR at discharge	Omitted due to collinearity		

Fine–Gray regression analysis was implemented to obtain hazard ratios, 95% CIs and *P*-values.

* Statistically significant.

CI: confidence interval; LAVVR: Left atrioventricular valve regurgitation; sHR: subdistribution hazard ratio.

Additionally, LVOTO relief surgeries were performed in 5 (2.3%) patients, 4 (1.8%) of these were done at the time of LAVV reoperation. Four (1.8%) patients required pacemaker implantation after cAVSD repair, and 3 (1.4%) of these were also performed at the time of LAVV reoperation. Two (0.9%) right ventricular outflow tract obstruction (RVOTO) relief operations were performed; both of these were individual reoperations. One (0.5%) aortic valve replacement due to regurgitation, 1 (0.5%) tricuspid valve replacement and 1 (0.5%) residual-VSD closure were performed as individual operations.

### Left atrioventricular valve regurgitation

Ninety-four (42.7%) patients developed moderate or more LAVV regurgitation during follow-up, of whom 16 (17.0%) had a repair with the double patch technique, 73 (77.7%) with the Australian single patch (*P* = 0.528) and 5 (5.3%) with the traditional single patch (*P* = 0.136).

The median time to moderate or more LAVVR development was 24.5 (5–257) days. The median time to moderate or more LAVVR was 7.0 (4.5–28.5), 46.0 (5–336) and 14.0 (9.6–160) days after the repair with the double patch, Australian single patch and traditional single patch techniques, respectively. There was no difference in median times to developing moderate or more LAVVR between the surgical techniques (*P* = 0.39).

**Figure 3:**
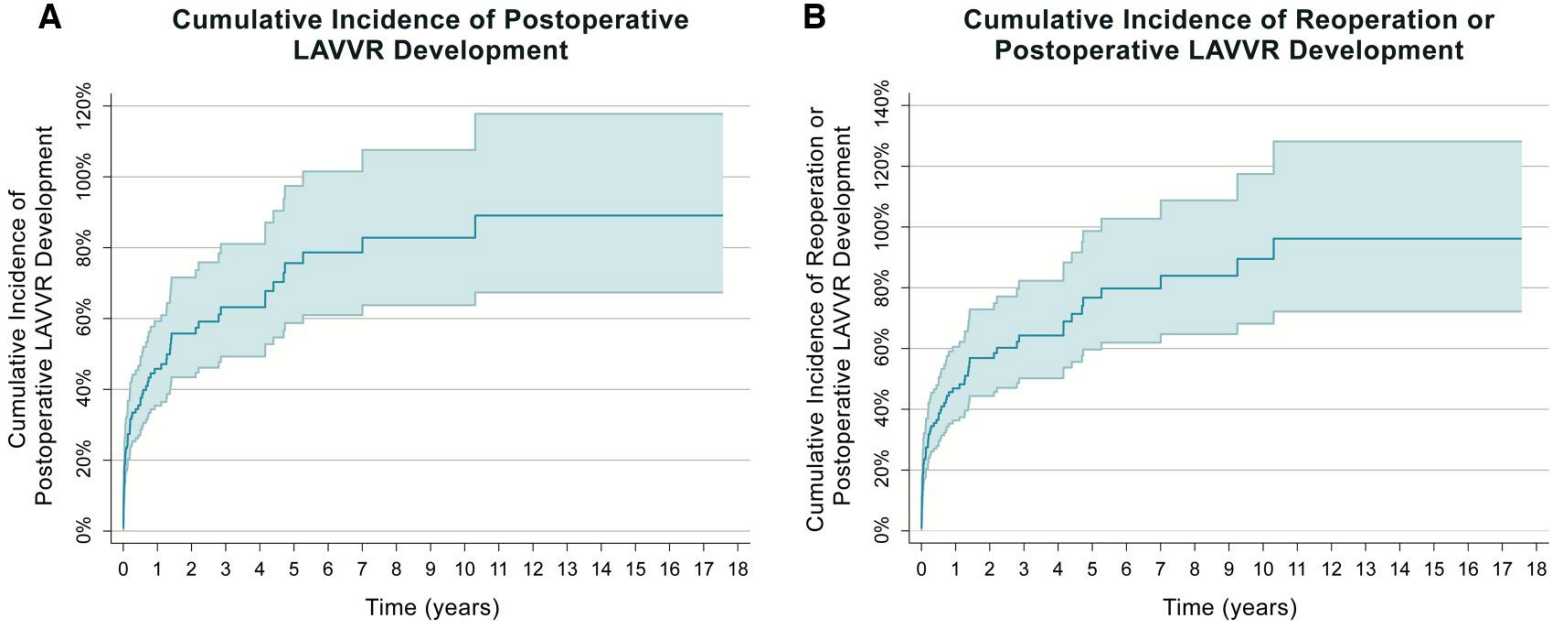
Cumulative incidence curves depicting freedom from postoperative moderate or more LAVVR development (left) and cumulative incidence of composite end point of developing moderate or more LAVVR or LAVV reoperation for all patients (right). LAVV: left atrioventricular valve; LAVVR: Left atrioventricular valve regurgitation.

The cumulative incidence estimates for LAVVR for all patients that underwent cAVSD repair were 0.76 (95% CI: 0.59–0.97) and 0.83 (95% CI: 0.64–1.08) at 5 and 10 years (Fig. 3). For patients who underwent repair with the double patch technique, the cumulative incidence was 0.72 (95% CI: 0.34–1.55) both at 5 and 10 years. After repair with the Australian single patch technique, it was 0.75 (95% CI: 0.57–0.99) and 0.83 (95% CI: 0.62–1.11), and after the repair with the traditional single patch repair, it was 1.28 (95% CI: 0.45–3.63) both at 5 and 10 years.

The cumulative incidence estimates for LAVV reoperation **or** LAVVR for all patients that underwent cAVSD repair were 0.77 (95% CI: 0.60–0.99) and 0.89 (95% CI: 0.68–1.17) at 5 and 10 years.

Of the 26 patients with immediate postoperative moderate or more LAVVR, 23 could be discharged. Among these 23, we also observed valve function improvement from moderate to mild in 9 (39.1%) patients at the time of discharge. However, of these 9 patients, 1 patient died, one other underwent reoperation and died later and another underwent reoperation.

At the last follow-up, 26 of the 176 (15%) survivors who did not have a reoperation for LAVVR had moderate or more LAVVR (5 after double patch repair, 20 after Australian single patch, 1 after traditional single patch repair).

Multivariable competing risk regression analysis using the Fine–Gray model identified post-repair LAVVR at any time on follow-up as the only risk factor for reoperation (*P* = 0.017),

### Length of hospital stay

The median length of hospital stay was 10 (10–22) days. By multivariable median regression analysis; prematurity (*P* = 0.012), surgical weight below 2.5 kg (*P* < 0.001), traditional single patch (*P* < 0.001) and immediate postoperative LAVVR (*P* < 0.001) were identified as predictors of prolonged post-repair hospital stay.

## DISCUSSION

The current study reports an extensive experience in definitive cAVSD repair with 3 different surgical repair techniques in 220 patients over a period of 21 years and delineates risk factors for adverse outcome.

Among the limitations of the current study its retrospective nature and the analysis period spanning 2 decades can be listed, as both could lead to the limitation of the accuracy of the collected data and the reflection of the advancements in operative and perioperative care.

### Operative and overall mortality

Survival rates ensuing surgical AVSD repair have improved drastically within the last 2 decades. Recent studies with similar patient characteristics report low to no operative mortality after cAVSD repair. However, most of these reflect 30-day mortality status rather than operative mortality defined by the STS [8–10]. In our cohort, our operative mortality was 4.6%, and there were only 3 (1.4%) mortalities within the 30 days following the repair. The contemporary ten-year survival rates for AVSD repair are above 90% [4]. Our 5- and 10-year survival rates were at 87.7% and 83.6%.

For cAVSD repair, the choice of repair technique has been an area of focus for years. Both the double patch technique and the Australian single patch techniques appear comparable in terms of mortality and risk of reoperations [4, 11, 12]. In our cohort, we observed a higher 10-year survival rate after cAVSD repair with the double patch technique than the Australian single patch technique even though, at the present time, this difference did not reach statistical significance. We currently prefer the double patch technique over the Australian single patch technique.

We identified 2 independent risk factors for overall mortality after cAVSD repair: prematurity and immediate moderate or more postoperative LAVVR. Prematurity has been uniformly flagged as a risk factor for mortality after repair surgery for congenital heart disease [13]. However, a significant association between prematurity and mortality after cAVSD repair has been rarely, if ever, reported. The current study demonstrates that the immediate post-operative presence of moderate or more LAVVR is the most important predictor of mortality. This finding is of relevance and should prompt all of us to try not to fail at first attempt or consider to perform an immediate second run operation to improve the valve in these cases. Historically, moderate or more LAVVR at hospital discharge was associated with the need for reoperation due to LAVVR, and reoperation has been associated with higher mortality [14]. Up to now, we had little information on the best approach on these patients.

ten Harkel *et al.* [15] in a review of 166 patients, argued that these patients need observation because up to 25% of patients who had immediate postoperative severe regurgitation may show an improvement of their regurgitation. However, in 50% of the patients, this improvement was transient with the need of a reoperation. We are, hereby, demonstrating that leaving a moderate or more LAVVR may influence mortality. We found that 40% of patients who are left with moderate or more LAVVR at the time of the operation would have a transient improvement but a third of those either died or were reoperated. It is yet unclear whether one should always go back to a second run procedure after the diagnosis of moderate or more AV valve regurgitation in the operation room. While we have recently adopted a somewhat aggressive strategy in going back on bypass, believing that a second run procedure carries a far smaller risk for the patients than leaving them with a significant burden of residual LAVVR, this strategy may not be beneficial in all hands and may depend on the various techniques used. The decision to go back on bypass remains an individual decision.

In Callahan's recent study [16] on AVSD patients from the STS database, an interatrial connection was associated with a lower long-term survival leading to the questioning of this common practice. In the current study, the fenestration of the atrial septum was performed in 61 patients, and a similar association was not observed. Although the number of patients in our study was smaller (220 vs 581), based on our experience with this relatively homogeneous group of patients from a single centre with a strict preoperative diagnosis (cAVSD versus AVSD) and almost uniform perioperative care (single centre versus 32 STS institutions), we continue performing a fenestration as needed.

### Left atrioventricular valve regurgitation development and left atrioventricular valve reoperation

Failing LAVV remains the Achilles' Heel of AVSD repair and accounts for the majority of reoperations which contributes substantially to the mortality and morbidity in these children [1, 4, 17–20].

Overall LAVV reoperation rate of 15% at the last follow-up in our study was similar to other studies in the literature [3, 21]. In the study from Xie *et al.* [19], in which they utilized both the double patch and the Australian single patch techniques, the LAVV reoperation rate was 13%. In a different study, Bakhtiary *et al.* [22] were able to reach a lower LAVV reoperation rate of 6%. In his cohort, all patients were operated on using the DP technique. In the subgroup of patients who underwent DP repair in our study, similarly an LAVV reoperation was needed in 6.3% of the patients.

The only risk factor for reoperation was moderate or more LAVVR in univariate analysis irrespective of the timing of the diagnosis. This association has also been shown previously [10, 15]. Only the presence of LAVVR at any time during follow-up was identified as a significant risk factor of reoperation. Whether the regurgitation was identified immediately postoperatively, at hospital discharge or later did not seem to influence that result.

LVOTO is another recognized complication after the surgical repair of cAVSD. In total, 5 (2.3%) LVOTO reoperations were performed in our cohort. This rate was similar to the reported rates in the literature [19, 23].

## CONCLUSION

The patients who undergo cAVSD repair remain subjected to a heavy burden of disease related to postoperative residual LAVVR. Immediate postoperative moderate or more LAVVR contributes significantly to overall mortality. Whether a second run of bypass can decrease this observed mortality should be investigated.

## Supplementary Material

ivae053_Supplementary_Data

## Data Availability

The data underlying this article will be shared on reasonable request to the corresponding author.

## Notes

Presented at the EACTS 36th Annual Meeting, 5-8 October 2022, Milan, Italy

## ABBREVIATIONS

AVSD: Atrioventricular septal defect
cAVSD: Complete atrioventricular septal defect
CI: Confidence interval
LAVV: Left atrioventricular valve
LAVVR: Left atrioventricular valve regurgitation
LVOTO: Left ventricular outflow tract obstruction
STS: Society of Thoracic Surgeons
