# Supplementary material for: Repair of the complete atrioventricular septal defect—impact of postoperative moderate or more regurgitation
Source: Interdiscip Cardiovasc Thorac Surg. 2024 Apr 3;38(4):ivae053. doi: 10.1093/icvts/ivae053 (PMC11055535; doi:10.1093/icvts/ivae053)
Supplement: ivae053_Supplementary_Data [file ivae053_supplementary_data.zip › Supplementary.docx]

**Supplement(s)**

**Operative Technique**

Surgical repairs were performed with aortobicaval cannulation, antegrade cardioplegia, and moderate hypothermia in both double-patch and Australian single-patch repair techniques. In the double patch technique, the VSD was closed with a patch that was sewn to the right side of the ventricular septal crest. It was followed by the fixation of the superior free caudal end of the patch to the atrioventricular valve tissue and the pericardial patch, which was then used to close the ASD. In the Australian patch technique, pledgeted sutures were placed similarly to the right side of the ventricular septal crest and then passed through the common atrioventricular valve and subsequently through the autologous pericardial patch, bringing the valvar axis down to the level of the ventricular crest. Cleft closure was left to the surgeons' discretion and was performed in 96% of the cases. Lastly, in the traditional single patch technique, the patch was passed through the existing cleft/indentation in the common atrioventricular valve leaflets which was incised up to the annulus. The patch was secured to the ventricular septal crest, the superior and inferior bridging leaflets and the atrial septal defect.

**Supplementary Table(s)**

**Table 1**

| **Patient # - GA and BW** | **Diagnosis** | **Operative Technique**  **Surgical age and weight** | **Cause of death** |
| --- | --- | --- | --- |
| 1. 34w- 2.25kg | Complete AVSD  Heterotaxy  Congenital duodenal atresia  s/p duodenostomy  Congenital complete heart block- s/p epicardial pacemaker imp. | Australian patch repair  11 months- 5.31kg | Sepsis and heart failure |
| 1. 35w- 2.66kg | Complete AVSD  Trisomy 21 | Australian patch repair  9 months 9 days- 7.45kg | Prolonged postoperative ECMO,  cardiopulmonary failure and multiorgan failure |
| 1. 37w-3.85kg | Complete AVSD  Trisomy 21 | Double patch repair  1 month and 12 days- 3.37kg | Prolonged postoperative ECMO,  cardiopulmonary failure and multiorgan failure |
| 1. 26w- 1.60kg | Complete AVSD  Trisomy 21 | Australian patch repair  5 months 22 days- 2.40kg | Cardiopulmonary failure, ECMO, renal failure, intracranial hemorrhage |
| 1. 38w- 2.86kg | Complete AVSD  Trisomy 21  s/p pulmonary artery banding and PDA closure | Australian patch repair  3 months 12 days- 2.81kg | Postoperative pulmonary hypertension and severe mitral regurgitation, ECMO, mitral reoperation, cardiac tamponade, therapy withdrawal |
| 1. 35w- 2.21kg | Complete AVSD  Trisomy 21 | Double patch repair  1 months 12 days- 3.37kg | Cardiopulmonary failure, ECMO, cerebral edema, microhemorrhage and seizures, therapy withdrawal |
| 1. 38w- 2.90kg | Complete AVSD  Trisomy 21 | Australian patch repair  10 weeks 4 days- 3.76kg | Postoperative cardiac arrest, ECMO, intracranial bleeding, therapy withdrawal |
| 1. 38w- 2.87kg | Complete AVSD  TAPVR | Australian patch repair  11 months 4days- 5.80kg | Respiratory failure, cardiac arrest |
| 1. 37w- 3.96kg | Complete AVSD  Hypoplastic aortic arch  Bicuspid aortic valve | Australian patch repair  5 months 2 days 4.30kg | Respiratory failure, cardiac arrest |
| 1. 40w-3.14kg | Complete AVSD  Trisomy 21  Pulmonary hypertension | Australian patch repair  1year 3 weeks 5.50kg | Respiratory failure |

**Supplementary table 1** depicts the characteristics of the patients who died within 30 days or before discharge after cAVSD repair.
